# Gingival Leukemic Infiltration in Chronic Lymphocytic Leukemia

**DOI:** 10.4274/tjh.galenos.2019.2018.0358

**Published:** 2019-11-18

**Authors:** Karima Kacem, Sami Zriba, Myriam Saadi, Raoudha Doghri

**Affiliations:** 1 Tunis El Manar University Faculty of Medicine, Department of Hematology, Tunis, Tunisia; 2 Aziza Othmana Hospital, Clinic of Clinical Hematology, Tunis, Tunisia; 3 Military Hospital, Clinic of Clinical Hematology, Montfleury, Tunisia; 4 Institute Salah Azaïz, Department of Pathology, Tunis, Tunisia

**Keywords:** Chronic lymphocytic leukemia, CD19, Leukemia

A 66-year-old female patient was referred for asymptomatic peripheral blood lymphocytosis. The blood smear showed 77% mature lymphocytes. Flow cytometry confirmed the clonality of the circulating B lymphocytes with positivity for CD19, CD5, and CD23. The patient was diagnosed with chronic lymphocytic leukemia (CLL), stage A, and a decision was made to watch and wait. Six years later, she was referred again for multiple adenopathy with splenomegaly. She reported a toothache that was worse upon biting, causing food restriction. Physical examination revealed multiple cervical lymphadenopathy, splenomegaly, and gingival enlargement with swollen margins and glossy texture (Figure 1). There were no exudates, necrosis, ulcerations, or active bleeding. Laboratory evaluation revealed a white blood cell count of 71,000/mm^3^, hemoglobin of 7.2 g/dL, and platelet count of 294,000/mm^3^. A biopsy of the gingiva showed infiltration by small lymphocytes expressing CD5 and CD20 positivity (Figures 2a and 2b). This typical pattern of CLL infiltration excluded a diagnosis of prolymphocytic leukemia or a transformation into aggressive lymphoma. The patient was treated with rituximab and chlorambucil for CLL, stage C. Gingival enlargement was less painful after one cycle, with reduction of the swelling. The patient was lost after the third cycle. Gingival hyperplasia due to leukemic infiltration is commonly observed in acute leukemia but is rare in CLL and represents an extranodal site. To our knowledge, only two prior cases were reported in the literature [1,2].

## Figures and Tables

**Figure 1:**
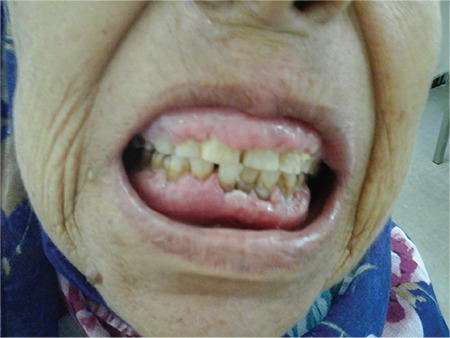
Physical examination revealed gingival enlargement with swollen margins and glossy texture.

**Figure 2:**
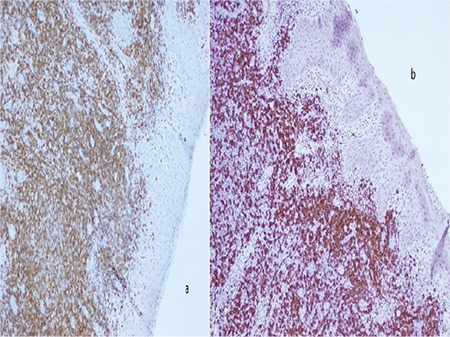
A biopsy of the gingiva showed infiltration by small lymphocytes expressing CD5 and CD20 positivity.
